# Simplified automatic method for measuring the visual field using the perimeter ZERK 1

**DOI:** 10.1186/s12938-016-0210-1

**Published:** 2016-07-25

**Authors:** Robert Koprowski, Paweł Kasprowski, Marek Rzendkowski

**Affiliations:** 1 Department of Biomedical Computer Systems, Faculty of Computer Science and Materials Science, Institute of Computer Science, University of Silesia, ul. Będzińska 39, 41-200 Sosnowiec, Poland; 2 Institute of Informatics, Silesian University of Technology, Akademicka 16, Gliwice, 44-100 Poland; 3 Individual Specialist Medical Practice, Gliwice, Poland

**Keywords:** Image processing, Measurement automation, Segmentation, Ophthalmology, Perimetry

## Abstract

**Background:**

Currently available perimeters have limited capabilities of performing measurements of the visual field in children. In addition, they do not allow for fully automatic measurement even in adults. The patient in each case (in any type of perimeter) has at his disposal a button which he uses to indicate that he has seen a light stimulus. Such restrictions have been offset in the presented new perimeter ZERK 1.

**Methods:**

The paper describes a new type of automated, computerized perimeter designed to test the visual field in children and adults. The new perimeter and proprietary software enable to carry out tests automatically (without the need to press any button). The presented full version of the perimeter has been tested on a head phantom. The next steps will involve clinical trials and a comparison with measurements obtained using other types of perimeters.

**Results:**

The perimeter ZERK 1 enables automatic measurement of the visual field in two axes (with a span of 870 mm and a depth of 525 mm) with an accuracy of not less than 1^o^ (95 LEDs on each arm) at a typical position of the patient’s head. The measurement can be carried out in two modes: default/typical (lasting about 1 min), and accurate (lasting about 10 min). Compared with available and known types of perimeters, it has an open canopy, proprietary software and cameras tracking the eye movement, automatic control of fixation points, light stimuli with automatically preset light stimulus intensity in the following ranges: 550–700 mcd (red 620–630 nm), 1100–1400 mcd (green 515–530 nm), 200–400 mcd (blue 465–475 nm).

**Conclusions:**

The paper presents a new approach to the construction of perimeters based on automatic tracking of the eye movements in response to stimuli. The unique construction of the perimeter and the software allow for its mobile use in the examination of children and bedridden patients.

## Background

Perimetry is a basic test associated with the quantitative evaluation of the visual field [1–3]. This is a subjective test [4]. Various factors influence its repeatability and reliability [5, 6]. This is mostly the cooperation of the patient with the camera, which requires concentration. The visual field tests are now done routinely during the eye examination performed by an ophthalmologist [7–9]. In special cases, they constitute an extremely valuable source of data (visual field). These are: glaucoma, diseases of the retina and the optic nerve, central nervous system damage [10, 11]. During the test and in all types of perimeters (PTS 920 from OPTOPOL Technology, AP-100-OPTOtech, Carl Zeiss Humphrey System 740 or Heidelberg Edge Perimeter), the patient sits in front of a semi-circular canopy resting the chin and forehead on the chin rest and the forehead rest [1, 12–15]. The patient looks at the fixation point with one eye [2, 3, 16]. Then the light stimuli appear automatically on the canopy. The brightness of the light stimuli is changed [17–19]. The patient’s task is to react to the light stimuli by pressing a button [20–22]. In this way, it is possible to precisely determine and analyse the sensitivity thresholds of the retina at various points with respect to the correct level. Additionally, on this basis, the maps of areas of good sensitivity of vision and its defects are plotted. The information associated with false-positive results is also stored [23, 24]. These are those cases when the patient presses a button signalling the presence of a light stimulus in its absence—it may be a deliberate act or a symptom of weariness. The test lasts for a few minutes and can be carried out in patients of any age [25–28]. The criterion for exclusion from the test concerns mentally ill people and pregnant women [26, 28].

Therefore, there are three problems in perimetry that have not been solved so far:

- Fully automatic measurement [29–31]—understood as a method that does not require any operator intervention and patient’s reaction to seeing a light stimulus;
- Limited use in young children;
- Deliberate introduction of erroneous results by the patient (by pressing the button routinely when the frequency of stimuli is constant).

The perimeter ZERK 1 described in this paper is devoid of the above-mentioned problems.

## Material

The developed software of the perimeter ZERK 1 was tested on the Intel^®^ Core i7 computer-3770 CPU 3.4 GHz, 10 GB of RAM. The eye movements were monitored by a system of EyeTribe and Genius Eye trackers. For instance, the EyeTribe tracker system is a Danish company producing modules for tracking the position of eyeballs. Tracking the eye movement involved a typical measurement consistent with the Declaration of Helsinki, and was performed in 20 healthy volunteers as part of routine applications of the EyeTribe tracker. On this basis, two perimeters were constructed. The first one, designed to test adults, had a chin rest and a forehead rest, and the other one, intended for measurements in young children, was movable and without the rest. The construction of the perimeter is described in detail in the following sections. Currently, the authors are at the stage of submitting an application to the ethics committee to get permission to test the entire system. Moreover, a utility model application has been filed at the European Patent Office. No research was carried out on humans as part of this work. All the results were obtained from routine tests performed with the EyeTribe tracker and (in the case of the full perimeter) tests on a phantom head (EYE Examination Simulator MB2A, Manikin size 42 × 21 × 38 cm, 2 kg)—in the case of testing the perimeter intended for children and adults.

## Methods

The perimeter ZERK 1 was created based on the two premises mentioned in the Introduction. The first one is the ability to perform measurement of the visual field in young children. Accordingly, the presented perimeter cannot have a closed canopy. The child needs to see the carer/parent, which reduces the fear of examination and prevents movements during the test. The second premise is the total automation of the examination. Owing to the built-in eye trackers, the patient’s reaction to the light stimulus is recorded. As a result, the patient does not press any button to signal that he sees the stimulus.

**Fig. 1:**
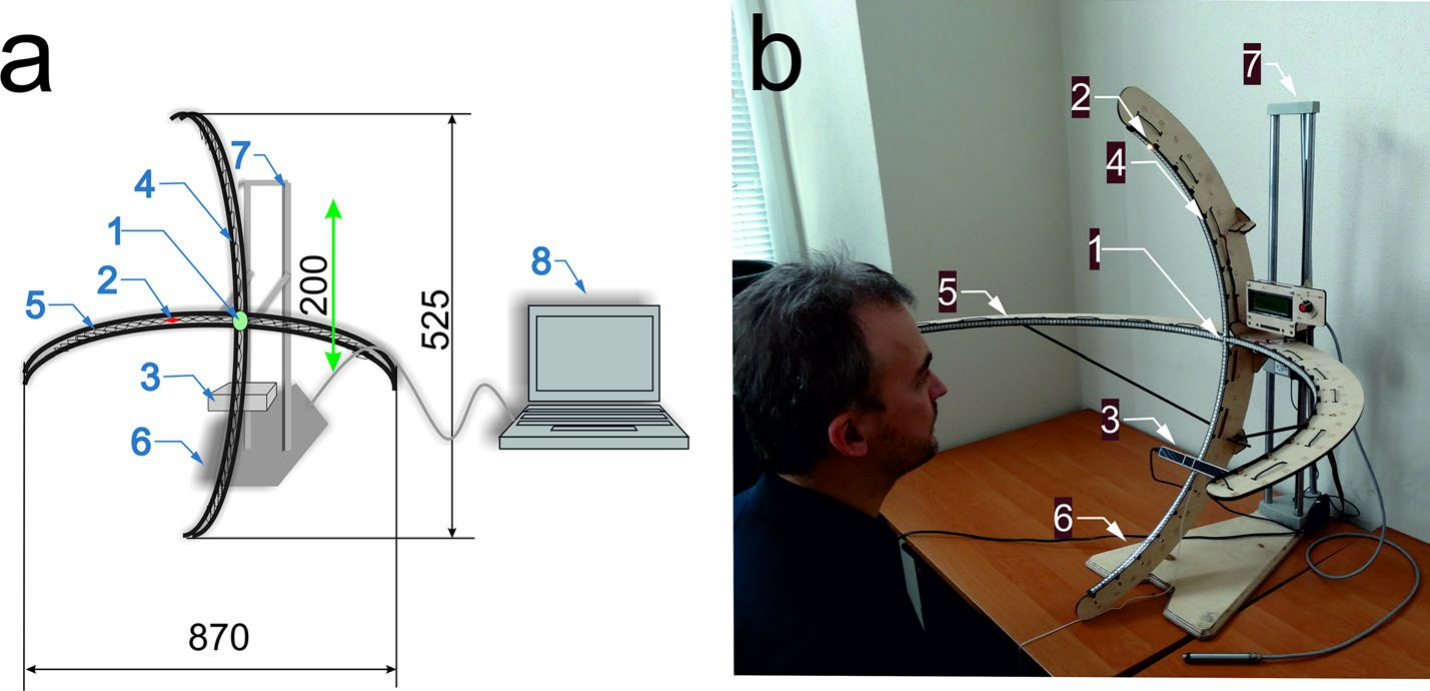
The perimeter ZERK 1: **a** Schematic diagram, **b** Image of the created perimeter. The numbers indicate: *1* central fixator, *2* LEDs, *3* eye tracking, *4* vertical arm, *5* horizontal arm, *6* base, *7* vertical guides, *8* computer with dedicated software

The perimeter ZERK 1 is made up of four basic elements—Fig. 1:

- Vertical and horizontal arms containing RGB LEDs (WS2812 Intelligent control LED integrated light source, power supply voltage 6.5 V, red 620–630 nm, 550–700 mcd, 20 mA, green 515–530 nm, 1100–1400 mcd, 20 mA, blue 465–475 nm, 200–400 mcd, 20 mA) placed at a linear distance from each other every 10 mm. The arms have a span of 870 mm (from the outermost left–right or up–down points) and a depth of 525 mm. The horizontal arm has a total of 190 LEDs, 95 LEDs arranged every 1° on each side. The vertical arm has 135 LEDs attached every 1°. In this way, the arms cover the full visual field;
- The fixation point placed in the main axis of the perimeter containing a colour marker. In the case of children, it is a colourful contour of a favourite character from a fairy tale or an animal. The size of the fixation point is about 20 × 20 mm;
- A system of EyeTribe trackers;
- A PC with windows operating system and developed software that enables to track the movement (location) of the eye in response to a light stimulus.

The task of the perimeter ZERK 1 is to preset light stimuli covering the full range of the visual field both vertically and horizontally. The analysis of the patient’s response to the light stimulus was carried out on the basis of the EyeTribe tracker which tracks the eyeball reaction. ZERK 1 can be removed from the base and placed directly next to a bedridden patient (child). The individual LEDs are controlled via USB as well as a microcontroller 89c2051 and USB converter. The test is carried out with the dedicated software in 2 modes:

- Simplified, fast mode—allowing for a 1-minute rough assessment of the visual field.
- Accurate, full mode—allowing for a 10-minute accurate assessment of the visual field.

**Fig. 2:**
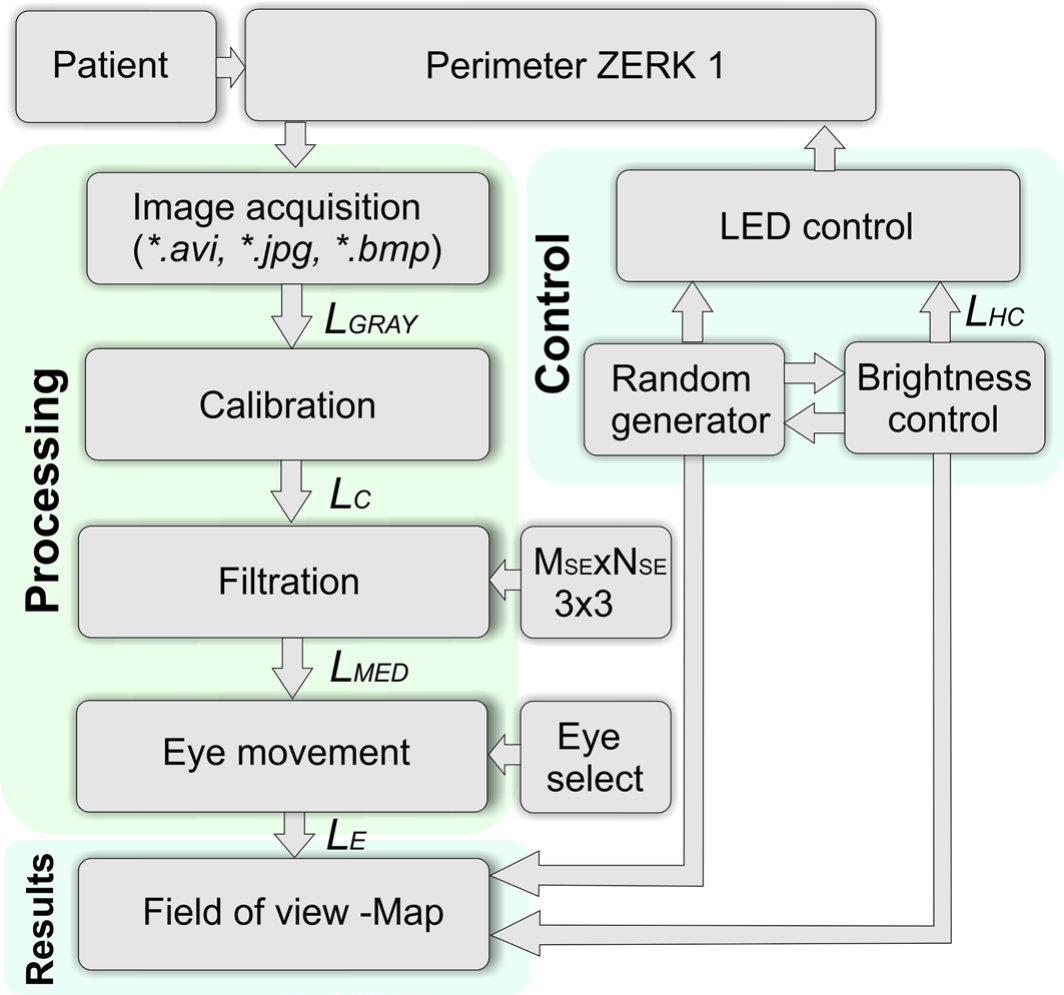
A simplified block diagram of the perimeter ZERK 1. The diagram shows the two main blocks. The first one is responsible for processing the eyeball movement. After acquisition, the image is calibrated (the head movement and uneven illumination are deleted). Next, the image is subjected to median filtering to detect the eye movement in the thus analysed eye movement image. The second block is responsible for controlling LED strips. It includes a microprocessor which controls the LEDs and brightness at random (pulse-width modulation). Explanations of variables *L*_*GRAY*_, *L*_*C*_, *L*_*MED*_, *L*_*E*_, *M*_*SE*_, *N*_*SE*_ and *L*_*HC*_ are presented in the text

In each test mode, the brightness (in the range of 550–700 mcd) and the order of stimuli are random. The interval between stimuli is also random and ranges from 0.5 to 5 s. The random position of the next stimulus and different length of the interval between stimuli prevents the patient from getting used to a scheme, prevents his weariness during the test, and thus reduces the number of false positive results (when the patient moved the eyeball out of habit although there was no stimulus). Figure 2 shows a simplified block diagram of ZERK 1. The two main blocks are presented in the diagram. The first one is responsible for processing the eyeball movement. After acquisition, the image *L*_*GRAY*_ is calibrated to the image *L*_*C*_ (the head movement and uneven illumination are deleted). Next, the image is subjected to median filtering [32–34] (image *L*_*MED*_) to detect the eye movement (*L*_*E*_) (after earlier covering of the left or right eye). The second block is responsible for controlling LED strips. It includes a microprocessor which controls the LEDs and brightness at random (pulse-width modulation—PWM). The merger of these two blocks results in a map (the patient’s visual field) created in an application developed in C++. Communication with the PC was realized via USB 2.0. It is two-way communication. The information about the eyeball movements is obtained from the eye tracker, whereas the information which LED, with what intensity, and where exactly on the perimeter is to shine, is returned. The methods of image analysis and processing shown in Fig. 2 provide the time of analysis of the response to a single stimulus of less than 10 ms, and are virtually negligible (do not affect significantly the overall result of the measurement time for the patient). It should be noted that the presented methods of analysis are one of the possible proposals for their implementation. Other methods of image analysis and processing can involve advanced morphological methods [29, 30], statistical methods [31, 32], or artificial intelligence methods and others [33, 35, 36].

## Discussion and results

**Fig. 3:**
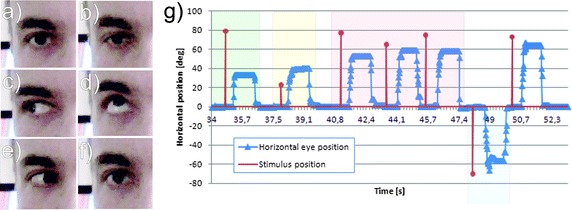
Examples of the extreme positions of the patient’s eye and the results obtained. The images from **a** to **f** are the subsequent positions of the left eye: starting with the central position, then down, right, up, left and back to the central position. **g** A sample graph of stimulus brightness changes (*red*) from 0 to 220 mcd for the position changes of the perimeter by the angle of 35° (*green background*), 40° (*yellow background*), 55°, 60° and 60° (*red background*) as well as −60° (*blue background*). The changes over time of the angular displacement of the left eye are marked in *blue*

The obtained results relate to the part of data analysis directly associated with the eye tracker. The data were collected retrospectively and relate to the assessment of the practical usefulness of data analysis methods and software. Figure 3 shows an example of the extreme positions of the patient’s eye and the results obtained. The images from Fig. 3a to f are the subsequent positions of the left eye: starting with the central position, then down, right, up, left and back to the central position. The part g shows a sample graph of stimulus brightness changes (red) from 0 to 220 mcd for the position changes of the perimeter by the angle of 35° (green background), 40° (yellow background), 55, 60 and 60° (red background) as well as −60° (blue background). The changes over time of the angular shift of the left eye acquired from the eye tracker are marked in blue. The response of the eye differs depending on the duration of the light stimulus. Because this is not the aim of the present study, this information has been omitted. However, it has been noted that there is a linear relationship between the light stimulus duration and the accuracy of localization (the angular accuracy of the eye movement toward the stimulus).

**Fig. 4:**
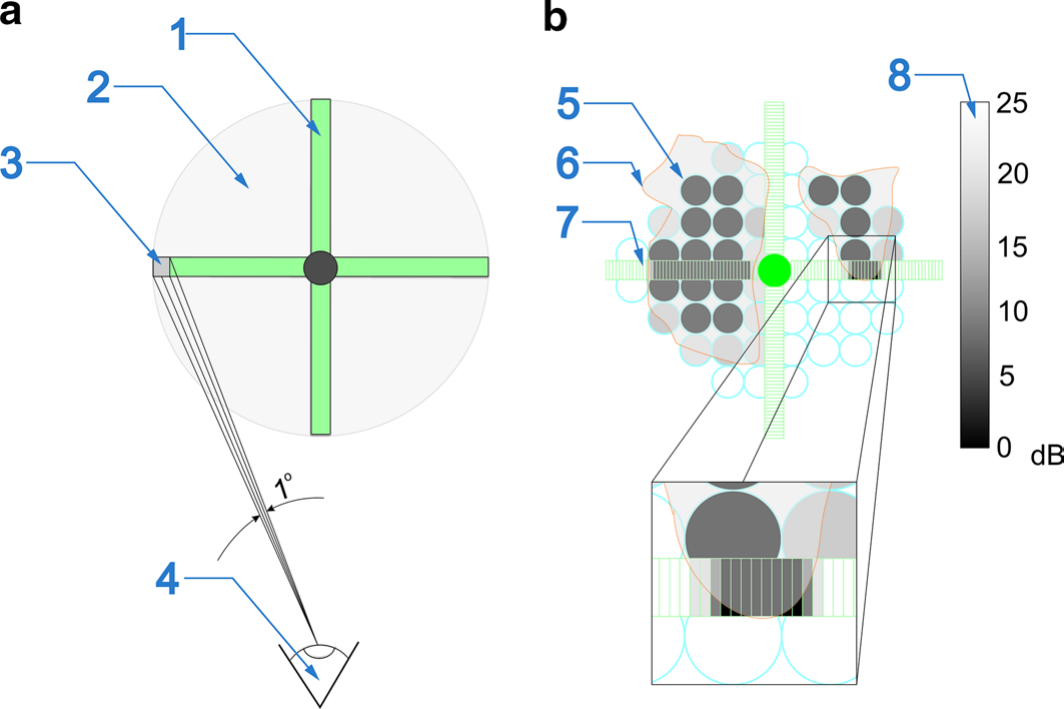
A comparison of the visual field for two types of perimeters: **a** Explanation of the angular accuracy of ZERK1 where *1* arms of ZERK1, *2* visual field, *3* the area of one LED, *4* position of patient’s eye; **b** Imposition of the results for the phantom where, *5* results obtained from the Heidelberg edge perimeter, *6* reference area for defects in the visual field, *7* results obtained using ZERK1, *8* *colour palette* of the threshold of eye sensitivity to stimuli expressed in dB

The full system of ZERK1 has been compared with the Heidelberg edge perimeter [4, 8, 22] and the artificial, preset map of the defects in the visual field. The selection of the Heidelberg Edge Perimeter resulted from the possibility of obtaining digital reference data concerning the measurement results. The angular accuracy of measurement with ZERK1 has been shown schematically in Fig. 4a. The preset defects in the visual field are shown in Fig. 4b together with the results obtained from the Heidelberg edge perimeter and ZERK1. The numerical comparison presented in Table 1 provides the following results related to the usability of ZERK1:

**Table 1:** Comparison of the basic parameters of the Heidelberg edge perimeter and ZERK1

Parameter	Heidelberg edge perimeter	ZERK1
Confirmation of seeing the stimulus	Manual (button)	Automatic
Measurement time (precise)	3 min	10 min
Measurement time (rough)	–	1 min
Measurement area	Central 10, 24 and 30° visual field	Central 10, 24 and 30° visual field—but only in two axes
Number of points	100	190 + 135
Analysis of the duration of the eyeball response to a stimulus	–	√
Possibility of measuring the visual field in children	–	√
Possibility of measuring the visual field in bedridden people	–	√
Angular resolution	1°	1°
Archiving results	√	√
Possibility of displaying a multicoloured stimulus	–	√
Sensitivity	From 0 to 25 dB	From 0 to 25 dB
Dependence of the results obtained on external lighting	–	√

- Fully automatic measurement—owing to the use of the system for tracking the eyeball movement;
- Quantitative results of the defects in the visual field horizontally and vertically with an angular accuracy of at least 1°;
- The possibility of obtaining additional parameters such as the speed of the patient’s eyeball response to a stimulus;
- Elimination of the human factor (cheating during measurement) owing to a random change of the stimulus location, a random change in its brightness and the analysis of the eyeball movements which do not result from the stimulus.

The limitations of ZERK1 should be also mentioned here:

- Measurement in only two axes—horizontally and vertically;
- The impact of the perimeter position on the results obtained (the absence of the chin rest is an option which results in difficulty in obtaining reproducible results—based on the measurements performed on the phantom, the error was ±10 % in the measurement of the distribution and the brightness level of the response to a stimulus);
- The need for fixation of the patient in the central position of the perimeter—and difficulty in staying focused on this point;
- The dependence on external illumination due to the open construction of the perimeter.

**Fig. 5:**
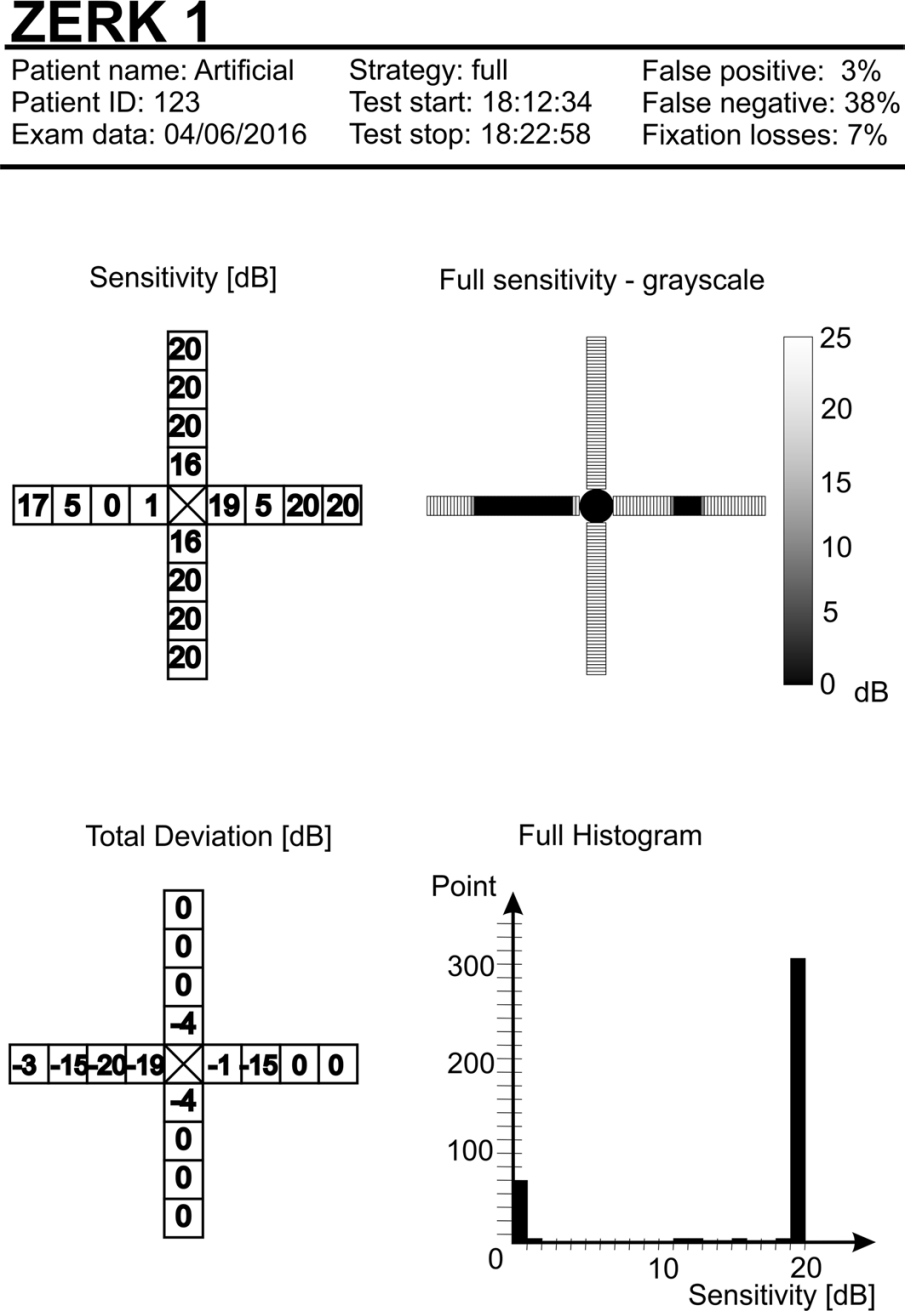
Example of a report (phantom). The *upper section* contains the logo and information such as the patient’s name, identification number, start and stop of the test, test type and the number of false positive and false negative results. The *lower section* provides the numerical results in a simplified version (*right*) and full version (*left*) in *gray* *levels* and as a histogram

An example of a report (phantom) from tests performed with ZERK1 is shown in Fig. 5. The upper section contains the logo and information such as the patient’s name, identification number, start and stop of the test, test type and the number of false positive and false negative results. The lower section (Fig. 5) provides the numerical results in a simplified version (right) and full version (left) in gray levels and as a histogram.

At the current stage, the authors plan thorough clinical trials in adults and children with various diseases, both those involving various types of defects in the visual field and those directly related to extra ocular muscle dysfunctions. They can reduce the possibility of carrying out measurements or exclude the patient from the test.

## Conclusion

The paper presents a new approach to the construction of a perimeter based on automatic tracking of eye movements in response to stimuli. Owing to this unique solution, the patient does not have to press any button to signal seeing the stimulus. The unique construction of the perimeter allows for its mobile use for the examination of children and bedridden patients. Currently, the authors intend to carry out detailed clinical trials of the created perimeter and compare the results with the results obtained using other perimeters. It is already known, on the basis of the presented preliminary results, that the new type of the perimeter will work perfectly in basic diagnostics of visual field (glaucoma, diabetes) in adults as well as in screening tests in children. The scope of diagnostic procedures will comprise monitoring the speed of the eyeball response and the assessment of sensitivity to a light stimulus. To conclude, the presented perimeter does not constitute competition for the known types of perimeters, it only completes the market in the area of fully automatic measurement, examination of bedridden patients and additional analysis of movement parameters (speed of response) of the eyeball.

## Abbreviations

AMD: age-related macular degeneration
ROI: region of interest
PWM: pulse width modulation
